# Accuracy and Self-Confidence Level of Freehand Drilling and Dynamic Navigation System of Dental Implants: An In Vitro Study

**DOI:** 10.7759/cureus.49618

**Published:** 2023-11-29

**Authors:** Mathew Mampilly, Leelamma Kuruvilla, Adham Abdulmajeed Tash Niyazi, Arun Shyam, Pallavi Ammu Thomas, Anzil S Ali, Fawaz Pullishery

**Affiliations:** 1 Oral and Maxillofacial Surgery, Esic Medical college and PG Institute, Bengaluru, IND; 2 Dentistry, Dr. Suzanne Caudry Implant Dentistry and Periodontics, Toronto, CAN; 3 Prosthodontics, Batterjee Medical College, Jeddah, SAU; 4 Conservative Dentistry and Endodontics, Kannur Dental College, Anjarakkandy, IND; 5 Public Health Dentistry, Tagore Dental College, Chennai, IND; 6 Public Health Dentistry, Royal Dental College, Palakkad, IND; 7 Community Dentistry and Research, Batterjee Medical College, Jeddah, SAU

**Keywords:** self-confidence, freehand drilling, dynamic navigation system, dental implant, angular deviation, accuracy

## Abstract

Background and objective: The impact of the experience of the clinician on learning a new skill or equipment was still an intriguing subject. The goal of this research is to determine the accuracy level of a dynamic navigation system to that of freehand drilling by expert and novice practitioners with varied levels of experience. Additionally, the duration of the surgical procedure and the self-confidence level of the surgeons were also evaluated.

Materials and methods: An analog impression of the patient was used to make 20 polyurethane simulation models of the maxilla. Five expert and five inexperienced surgeons prepared the site and placed the implants at random on ten models each. Two different techniques were used to insert dental implants: freehand and dynamic navigation systems. Dental implants were placed in Group 1 utilizing a computer-assisted dynamic navigation device. The implants in Group 2 were secured using free-hand drilling. The dental implants were inserted first in the maxillary right first molar, then in the maxillary right lateral incisor, and the maxillary left second premolar. Preoperative and postoperative CBCT scans were superimposed by employing the Evalunav software and contrasted. The coronal 3-D, apex 3-D, apex vertical depth, and angular deviations for both procedures were evaluated. A pre-tested self-confidence questionnaire was also administered to assess the self-confidence of the practitioners. The duration of the surgical time was also documented for each strategy. The t-test was used to measure the difference in accuracy and confidence levels between freehand and dynamic navigation systems among expert and novice surgeons using SPSS software (IBM Corp., Armonk, NY, USA).

Results: A total of 60 implants were used (three insertion sites, two methods, and 10 practitioners). Each of the five expert and novice clinicians implanted 15 implants (five models each). Except for entry 3-D, there was a statistically significant difference between the two approaches in all of the primary outcome variables. The apex 3-D (5.89±1.08 mm) and apex vertical (2.08±1.27 mm) dimensions of the dynamic navigation system were significantly smaller than those of the freehand drilling approach (p<005). Dynamic navigation and freehand drilling had angular deviations of 7.16±1.76ᵒ and 9.06±2.18ᵒ, respectively (p=0.0004). The apex vertical deviation was reduced in the navigation technique (2.07±1.5 mm) than in the freehand drilling (2.86±1.4 mm) by experienced practitioners (p=0.04). The difference in time between the two procedures was determined to be statistically highly significant (p<0.001) by both expert and novice surgeons. Furthermore, when contrasting with experienced practitioners, novice practitioners had an overall increase in surgery time (p<0.001) for both approaches.

Conclusion: The current in vitro study found that the dynamic navigation system enables more accurate implant placement than the freehand drilling technique, irrespective of the experience of the surgeons. However, this technique appears to benefit novice practitioners more, as they can profoundly minimize their deviations while accomplishing results comparable to those of expert surgeons.

## Introduction

With computer-guided implant surgery, clinicians can accurately duplicate the anticipated implant position during the surgical procedure [1]. During the procedure, the dynamic navigation enables real-time tracking of implant bed preparations. The dentition is registered and the computed tomography (CT) or cone-beam CT (CBCT) finding is reconstructed, and the oral surgeons can monitor the exact location of the surgical drills on the CBCT reconstruction using specialized software and tracking methods for observing the rotary motion of the surgical zone and instruments [2]. As a result, any deviations in the implant and drill could be tracked and addressed. Furthermore, it allows us to change the virtual contemplating throughout the procedure.

A substantial number of implants for dentistry are now inserted using freehand drilling, with no aid from computer three-dimensional (3-D) simulation. The surgeon makes an osteotomy with only adjoining and opposing teeth as a guide for position and then inserts the implant freehand [3]. It is projected that roughly 7% of challenges are caused by incorrect alignment of implants. Furthermore, it has been found that nearly 20% of the implants had improper distances to adjacent teeth or implants and that 30% of the implants had perforation of surrounding structures. In reality, accomplishing the optimum 3-D implant placement reduces risks associated with surgery such as sinusitis, nerve damage, hemorrhage, or aesthetic issues including buccal dehiscence caused by the resorption of the buccal plate, prosthetic concerns such as challenges placing a restoration, and marginal bone loss [4,5]. It also relieves the psychological and ergonomic stress of the professionals during the procedure [6,7]. Computer-driven surgery is typically used in intricate situations when anatomical circumstances, notably the closeness of the inferior alveolar nerve, necessitate extremely precise surgery with the goal to avoid injuries [8].

Accuracy is regarded as an important metric in assessing the clinical relevance of various implant surgical techniques. The discrepancy in location or degree of angulation between the treatment plan and installation is characterized by the accuracy of a computer-guided process, and flaws can arise from acquiring images to surgical implant placement. An additional CBCT or multislice CT scan is employed to figure out the compatibility between the intended and implanted implant placements, facilitating the fusion of the preoperative planning and postoperative implant positions. There is always some difference between virtual planning and real implant placement in vivo [9]. However, a dynamic navigation system necessitates extensive training and expertise [10]. The expensive nature and technological complexities associated with navigational devices have limited their application. Until lately there were few clinically viable dynamic navigation devices for dental implants [11]. The impact of the experience of the clinician on learning a new skill or equipment was still an intriguing subject [12]. The goal of this research is to determine the accuracy level of a dynamic navigation system to that of freehand drilling by expert and novice practitioners with varied levels of experience. Additionally, the duration of the surgical procedure and the self-confidence level of the surgeons were also evaluated.

## Materials and methods

The in vitro study was performed in accordance with the Helsinki Declaration on the Conduct of Medical Research. The Institutional Ethical Research Committee at Batterjee Medical College, Jeddah endorsed the study (RES-2023-0094). Two different techniques were used to insert dental implants: freehand and dynamic navigation systems (Navident, ClaroNav Technology, Toronto, ON, Canada). The sample size was determined using G*Power v.3.1.3 (Heinrich-Heine Universität, Germany), with angular deviation as the major outcome variable. Measurements on mean angulation deviation were obtained from an earlier published work [13]. An alpha value of 0.05 and a statistical power of 80% were estimated. The sample size estimation determined that 60 implants were required for the current study (30 implants for each technique).

Dental implants were placed in Group 1 utilizing a computer-assisted dynamic navigation device (Navident, ClaroNav). The implants in Group 2 were secured using free-hand drilling. In order to simulate a real-life situation, the dental implants were inserted first in the maxillary right first molar, then in the maxillary right lateral incisor, and finally in the maxillary left second premolar. A website-produced random sequence was utilized, and each implant site was randomly assigned to either of the two techniques. To ensure allocation concealment, investigators did not notify the group that each implant was designated until shortly before the drilling process began after elevating the flap. By allocating distinct examiners and professionals to various assigned duties, participant bias was reduced. The experienced oral surgeon with clinical experience of over five years in implant dentistry was selected. The surgeon who performed the implant installation did not participate in surgical planning or assessment of implant insertion accuracy. The examiner who measured the outcome measures was not aware of the surgical approach employed during the simulation procedures.

Preparation of the models

Partially edentulous healthy individuals were selected for the study. An analog impression of the patient was used to make 20 polyurethane simulation models of the maxilla. CBCT i-CAT (I-CAT, Imaging Science International, Hatfield, PA, USA) was used for imaging each model. The models were outfitted with three metallic fiducial sphere indicators before scanning to allow for precise superimposition of preoperative and postoperative CBCT imaging. A metallic stent and a dual-arm computerized tomography indicator (ClaroNav) were set up on the model preceding scanning. The Navident software (ClaroNav) was used to virtually plan crowns and implants. Five expert and five inexperienced surgeons prepared the site and placed the implants. Oral surgeons with above five years of surgical expertise in implant dentistry were considered experienced practitioners, while oral surgeons with only six months of clinical competence in implant dentistry were considered novice clinicians. Prior to the investigation, all practitioners underwent typical hands-on instruction for virtual planning with implant procedures. Therefore, both had previous experiences with model simulations and dynamic navigation systems. Models have been allocated at random to both study groups.

Pre-operative CBCT and virtual treatment planning

**Figure 1:**
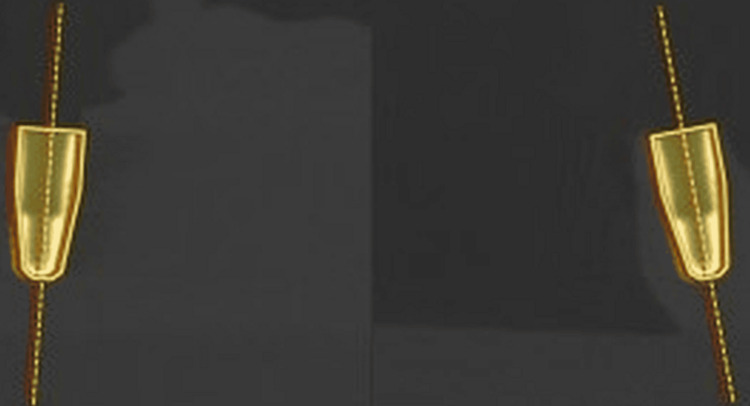
Post-implant placement CBCT scans CBCT: Cone-beam computed tomography

Preoperative CBCT scans were performed before the simulation procedures. The scanning parameters were outlined as follows: 8x10 cm field of view, 110 kV, 0.25 mm voxel size, 360ᵒ rotation, 9 seconds exposure time, 94 kV tube voltage, and 7.2 mA tube current. 3-D virtual digital planning software was used for planning the implant placement. The STL file of the maxilla was registered with the Digital Imaging and Communications in Medicine data (DICOM) of the preoperative CBCT reconstruction process. Considering the zygomatic anatomy-guided technique, the DICOM details were transferred into the dynamic navigation (Navident) treatment planning program [14]. The software enables the creation of a simulated wax-up of the suggested prosthesis and is capable of moving effortlessly into the implant location and adjusting the dimensions as necessary. After the simulated wax-up was wrapped up, implant planning was commenced. The dental implants were designed in a standardized manner, with the ability to modify their dimensions (Figure 1).

Prior to and following implant insertion, the handpiece and drills were calibrated in accordance with the Navident workflow procedure. Following implant insertion, a postoperative CBCT scan was performed and projected on the intended preoperative CBCT scan by employing the Evalunav (ClaroNav) software. Following superimposition, the originally intended and mounted implant placements were contrasted, and the coronal 3-D, apex 3-D, apex vertical depth, and angular deviations for both procedures were evaluated.

Model surgery

To mitigate the fatigue of the investigators, surgical procedures were conducted in a randomized order across three days, with five model surgeries (15 implants) each day. The models were anchored before the procedures to prevent intraprocedural displacement. All osteotomies were performed with an 800-rpm speed of rotation of the drill and external conditioning. In Group 2, the implant osteotomies were performed using freehand drilling while contemplating the general requirements for the placement of three implants on each model [15]. Each operator placed the implants in ten separate maxillary models. For each maxillary model, three implant locations, namely 16, 12, and 25, were proposed. On a typical dental mannequin head with rubber cheeks, implants were planned (Root form tapered implant 4.2mm x 10mm in dimensions) and placed utilizing a freehand drilling and dynamic navigation technique.

Outcome variables

The entry 3-D deviation in the coronal surface of the alveolar ridge, apex 3-D deviation at the apex of the implant, vertical depth deviation at the apex, and angular deviation were the key outcome factors. A pre-tested self-confidence questionnaire was also administered to assess the self-confidence of the practitioners on a rating system of 1 to 30 [16]. The duration of the surgical time was also documented for each strategy as a secondary outcome variable.

Statistical analysis

IBM SPSS Statistics for Windows, version 25.0 (IBM Corp., Armonk, NY, USA) was used to analyze the data. The Shapiro-Wilk test was used to determine the normality, and a p-value of less than or equal to 0.05 was deemed statistically significant. The t-test was applied to measure the difference in accuracy and confidence levels between freehand and dynamic navigation systems among expert and novice surgeons.

## Results

A total of 60 implants were used (three insertion sites, two methods, and 10 practitioners). Each of the five expert and novice clinicians implanted 15 implants (five models each). Two implant sites were perforated at the apical region of the palatal bone as a result of freehand drilling by novice practitioners. Table 1 shows the mean deviation of the outcome variables from dynamic navigation and freehand drilling.

**Table 1:** Distribution of the outcome variables between the dynamic navigation and freehand drilling

Variable	Entry (3-D) (Mean±SD mm)	Apex (3-D) (Mean±SD mm)	Apex vertical (Mean±SD mm)	Angulation (Mean±SD in degrees)	Surgical time (seconds)
Dynamic navigation (n = 30)	5.34±1.45	5.89±1.08	2.08±1.27	7.16±1.76	570±1.6
Freehand drilling (n = 30)	6.19±3.14	6.95±2.12	3.06±2.11	9.06±2.18	319±2.99
P-value	0.18	0.02	0.03	0.0004	<0.0001

Except for entry 3-D, there was a statistically significant difference between the two approaches in all of the primary outcome variables. The apex 3-D (5.89 ± 1.08 mm) and apex vertical (2.08 ± 1.27 mm) dimensions of the dynamic navigation system were significantly smaller than those of the freehand drilling approach (p < 005). Dynamic navigation and freehand drilling had angular deviations of 7.16 ± 1.76ᵒ and 9.06 ± 2.18ᵒ, respectively (p = 0.0004). The practitioners took 570 ± 1.6 seconds and 319 ± 2.99 seconds for dynamic navigation and freehand drilling, respectively (p < 0.0001). All of the primary outcome variables were significantly reduced with the dynamic navigation system than with the freehand procedure.

When the outcomes of the experience of the surgeons were analyzed, the inexperienced clinician demonstrated a greater degree of accuracy for all of the outcome variables evaluated (p < 0.05). With the dynamic guidance system, the experienced surgeons had optimal angulation than the less-experienced surgeons (p = 0.02). In a comparable manner, the apex vertical deviation was reduced in the navigation technique (2.07 ± 1.5 mm) than in the freehand drilling (2.86 ± 1.4 mm) by experienced practitioners (p=0.04). The novice and experienced practitioners spent considerably more time using the navigation technique than freehand drilling (p < 0.001) (Table 2).

**Table 2:** Comparison of the outcome variables of the dynamic navigation and freehand drilling techniques within the practitioners

Variable	Entry (3-D) (Mean±SD mm)	Apex (3-D) (Mean±SD mm)	Apex vertical (Mean±SD mm)	Angulation (Mean±SD in degrees)	Surgical time (seconds)
Experienced surgeons (n=5)					
Dynamic navigation	5.55±1.81	5.74±0.77	2.07±1.5	7.16±2.01	547±1.7
Freehand drilling	5.98±1.69	6.36±1.83	2.86±1.4	8.4±1.92	298±1.9
p-value	0.34	0.09	0.04	0.02	<0.0001
Novice surgeons (n=5)					
Dynamic navigation	5.13±1.1	6.04±1.39	2.09±1.03	8.06±1.5	593±1.5
Freehand drilling	6.4±2.9	7.53±2.41	3.26±2.81	9.71±2.44	340±4.07
p-value	0.03	0.005	0.04	0.002	<0.0001

The self-confidence questionnaire demonstrated an insignificant variance of competency in the dynamic navigation method between experienced and novice practitioners (p = 0.11). However, novice practitioners stated that employing the navigation technology enhanced their clinical competence and accuracy level more than freehand drilling (Table 3), with a statistically significant difference (p = 0.03).

**Table 3:** Mean self-confidence score of the dynamic navigation and freehand drilling techniques between the practitioners The total range of the mean self-confidence score is approximately 4.8 to 6.4. A score of 5: Highly confident and skilled, a score of 4: Moderately confident and skilled, a score of 3: Moderate confidence and skill, a score of 2: Low confidence and skill, a score of 1: Very low confidence and skill, a score of 0: No confidence or skill

Variable	Dynamic navigation		Freehand drilling	
	Experienced (n = 5)	Novice (n = 5)	Experienced (n = 5)	Novice (n = 5)
Level of confidence during the procedure	5	4	4	3
Level of surgical skill during the procedure	5	4	5	4
How worried were you during the intervention?	4	4	4	3
How anxious were you during the intervention?	4	4	4	3
Would you like to avoid this procedure altogether?	5	4	4	3
How independent were you in planning of the implant placement and performing the intervention?	5	4	5	4
Total	28	24	26	20
Mean score	5.6±0.81	4.8±0.6	5.2±0.51	4±0.9
P-value	0.11		0.03	

## Discussion

The present in vitro study was conducted to evaluate the accuracy of the dynamic navigation dental implant system to that that of conventional freehand drilling. The goal of a navigation system in implantology is to reduce the divergence of the implant position from preoperative planning by using real-time drilling and implant insertion monitoring. The navigation system considerably improved the accuracy levels of novice professionals with respect to the entrance 3-D, apex 3-D, apex vertical, and angulation.

Several investigations have looked into the use of computer-aided navigation systems in the placement of zygomatic dental implants. The results of Xiaojun et al. [17] showed a mean coronal entrance point of 1.35 ± 0.23 mm, an apical endpoint of 1.57 ± 0.243 mm, and an angular variance of 4.11 ± 0.9°, which were lower than those reported in the current investigation for dynamic navigation technique. However, Rueda et al. [18] found that navigation implants had a mean coronal entry, apical endpoint, and angular deviation of 5.43 ± 2.13 mm, 4.92 ± 1.89 mm, and 7.36 ± 4.12°, which was comparable to the current study outcomes. Stünkel et al. [19] demonstrated improved precision for pilot drilling guides in average coronal and apical deviations and greater accuracy for angular variance for dynamic navigation. The disparity in results could be attributed to the use of distinct drilling or guiding techniques, varied scanning methodologies for post-operative measures, and varied implant sites in either of arch.

It was shown that the vertical and angular variations of novice practitioners were greater than those of expert surgeons [5]. Hoffmann et al. noticed statistically significant differences in accuracy level between implants positioned using dynamic navigation systems and those placed by freehand, with mean angular variations of 4.2 ± 1.8° and 11.2 ± 5°, respectively, which was consistent with our findings [20]. Tao et al. [21] demonstrated that radio-diagnostic technology influences the dynamic navigation approach, particularly at the apical deviation level, with CBCT attaining greater accuracy levels than standard multislice CT. Thus, dynamic navigation technologies have proven to be highly beneficial in communicating the treatment plan to the patient and preventing difficulties associated with the insertion of dental implants for the repair of profound maxillary atrophy and maxillary deformities [18].

There is a varying learning curve for establishing competency with a dynamic navigation device. According to clinical studies, 15 to 125 case scenarios are needed, contingent upon the operation and the usage of surgical simulation equipment, before the clinicians achieve expertise with novel surgical techniques [22]. Wang et al. investigated the learning curves of two dynamic navigation techniques that approximated each other following 12 dental implants and eventually harmonized after 27 implants [23]. Cassetta et al., on the other hand, found no impact on the learning curve for dental implants inserted with a static navigation device [24]. Furthermore, the potential variance in accuracy during surgery between freehand drilling, dynamic, and static navigation systems was emphasized in a concurrent in vitro study by Chen et al. and was claimed that the dynamic navigation system displayed greater accuracy at the apical and angular levels [25].

In the current investigation, regardless of approach, novice surgeons took considerably greater time to complete the surgery, which might be ascribed to the competency and surgical expertise of the practitioners. The navigation strategy gave inexperienced practitioners an immense amount of confidence, which was in accordance with another study [13].

A considerable amount of attention and longer are necessary from both the novice and experienced surgeons for a favorable outcome which in turn coincides with a learning curve [18]. The point-based registration technique used in navigation systems for determining fiducial markers (black-and-white tags) is susceptible to errors, such as fiducial localization error, which is characterized by the erroneous recognition of the fiducial markers by the software depending on the image voxel size along with the dimensions of the fiducial indicators. Furthermore, the possibility of faults including fiducial registration error (the root mean square difference between adjacent fiducial markers) and target registration error should also be considered [17]. The inconsistency between the coordinator of the navigation tool and the associated investigator of the surgical field, which is critical for executing the surgery securely and precisely, is referred to as trial registration error. As a result, implanted bone-anchored screws have been demonstrated to be the most precise fiducial indicators available and are considered to be the benchmark for point-to-point registration [26].

The clinical applicability of dynamic navigation systems is constrained due to higher expenses a steep learning curve, and the potential of incorrect positioning of implants attributable to system inaccuracy related to either registration or calibration phases, particularly in totally edentulous individuals. Furthermore, a great deal of evidence evaluating navigation system precision primarily relies on in vitro investigations, and clinical trials are relatively sparse. As a result, additional clinical trials are needed to evaluate the fact that the implant positioning accuracy and time efficiency endure in a real-world clinical setting [13].

The current in vitro investigation could impact the generalizability of the results, particularly those that can be influenced by clinical factors. The current study, on the other hand, exhibits excellent internal validity while controlling for various confounding factors that cannot be adjusted in a true clinical setting. Indeed, all of the preclinical simulated models, ambient lighting, surgical drilling devices, implant systems, implant dimensions, and CBCT software components employed during presurgical planning have been rendered identical to analyze the influence of experience on system accuracy without potential confounders. Another element that should be examined in future studies is the relationship between the accuracy of the implant position between the maxillary and mandibular arch.

The study has some limitations such as the research was conducted in a controlled laboratory setting using polyurethane simulation models of the maxilla. While this provides a standardized environment for assessing accuracy, the findings may not fully represent real-world clinical conditions with variations in patient anatomy, soft tissue management, and clinical challenges. The small sample size may limit the generalizability of the results to a broader population of clinicians. Self-confidence is a subjective measure and can vary among individuals, potentially introducing bias. Further research with larger sample sizes, diverse clinical scenarios, and long-term assessments is needed to provide a more comprehensive understanding of the topic.

## Conclusions

The current in vitro study found that the dynamic navigation system enables more accurate implant placement than the freehand drilling method, irrespective of the experience of the surgeons. However, this technique appears to benefit novice practitioners more, as they can profoundly minimize their deviations while accomplishing results comparable to those of expert surgeons. The inevitable advancement of analog 2-D imaging to digital 3-D imaging and diagnostics has resulted in a better knowledge of the complexities of surgical implant procedures and prostheses. The dynamic navigation enables the surgeon to effectively execute the digital implant treatment regimens.
